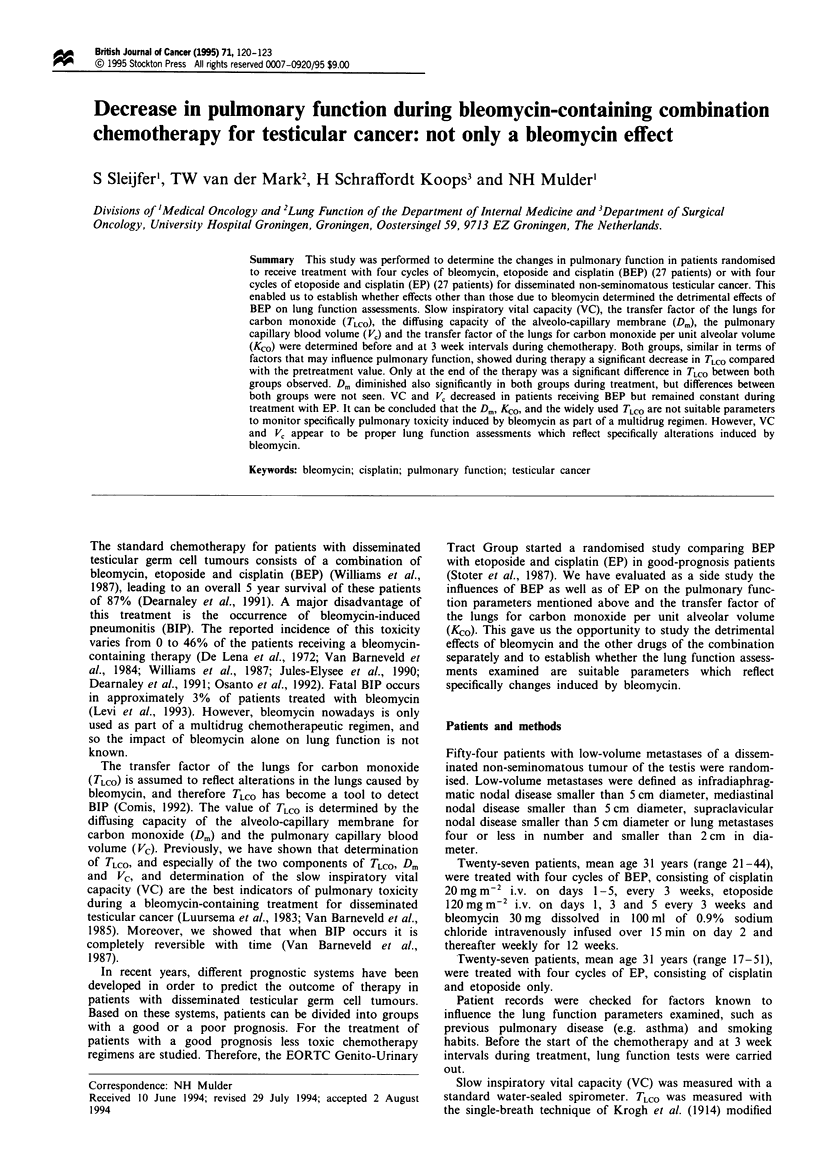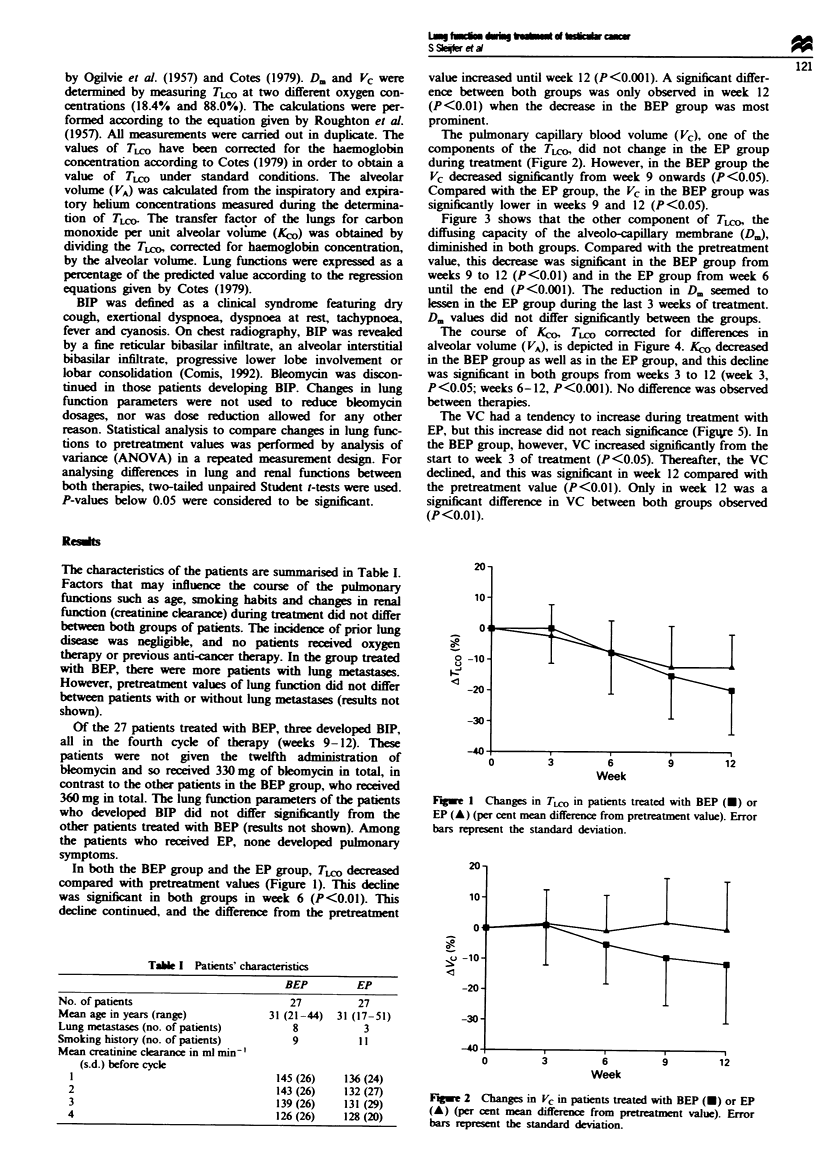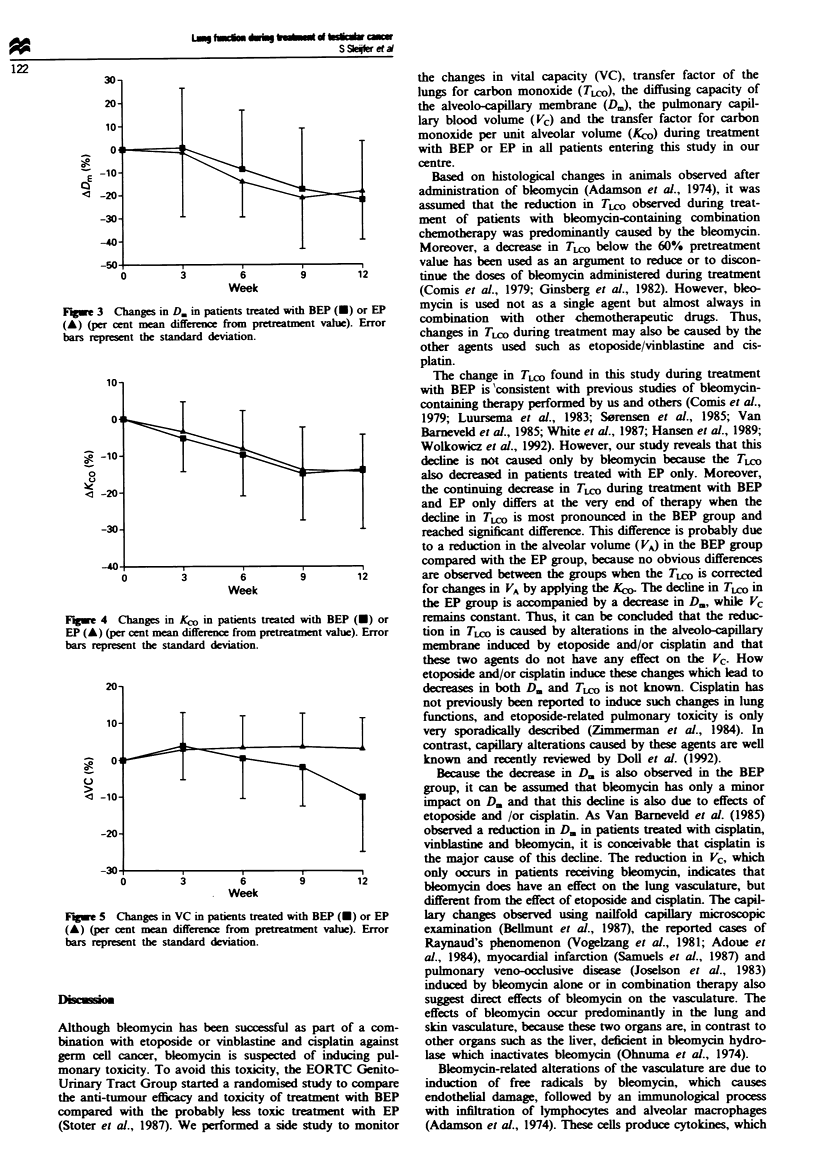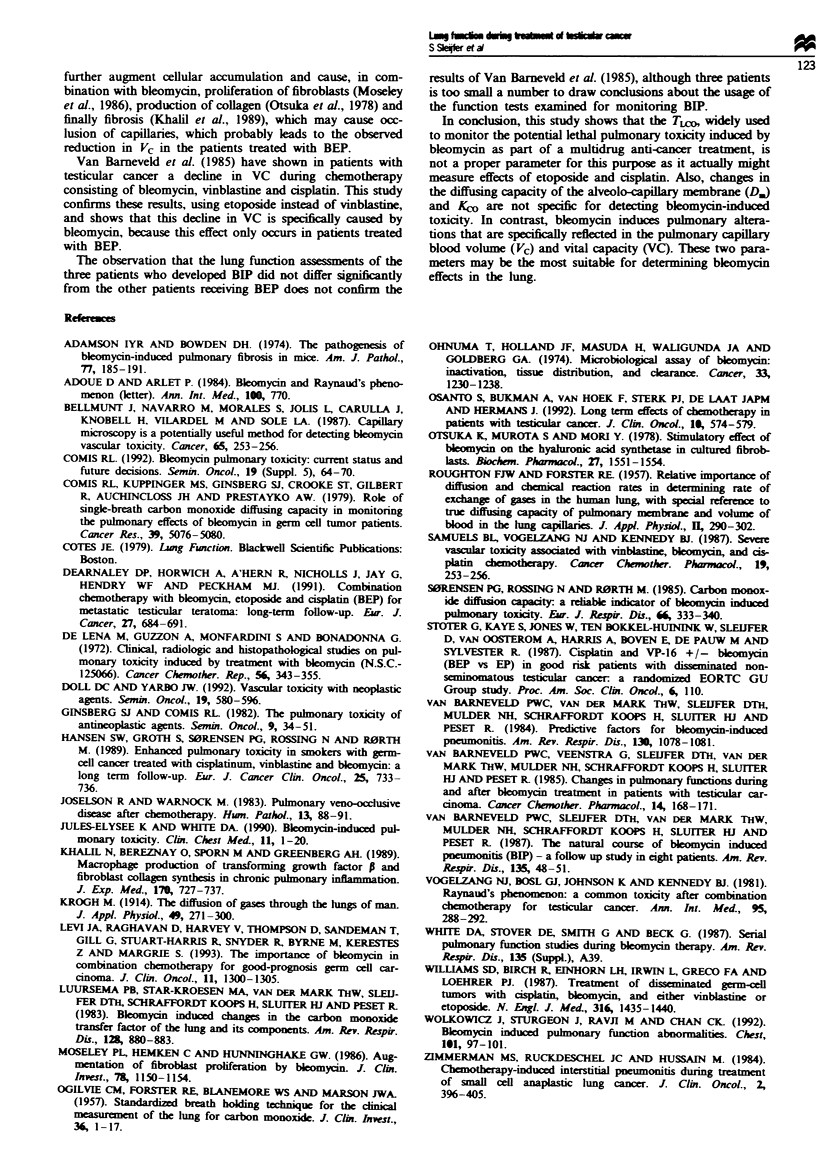# Decrease in pulmonary function during bleomycin-containing combination chemotherapy for testicular cancer: not only a bleomycin effect.

**DOI:** 10.1038/bjc.1995.24

**Published:** 1995-01

**Authors:** S. Sleijfer, T. W. van der Mark, H. Schraffordt Koops, N. H. Mulder

**Affiliations:** Divisions of Medical Oncology, University Hospital Groningen, The Netherlands.

## Abstract

This study was performed to determine the changes in pulmonary function in patients randomised to receive treatment with four cycles of bleomycin, etoposide and cisplatin (BEP) (27 patients) or with four cycles of etoposide and cisplatin (EP) (27 patients) for disseminated non-seminomatous testicular cancer. This enabled us to establish whether effects other than those due to bleomycin determined the detrimental effects of BEP on lung function assessments. Slow inspiratory vital capacity (VC), the transfer factor of the lungs for carbon monoxide (TLCO), the diffusing capacity of the alveolo-capillary membrane (Dm), the pulmonary capillary blood volume (Vc) and the transfer factor of the lungs for carbon monoxide per unit alveolar volume (KCO) were determined before and at 3 week intervals during chemotherapy. Both groups, similar in terms of factors that may influence pulmonary function, showed during therapy a significant decrease in TLCO compared with the pretreatment value. Only at the end of the therapy was a significant difference in TLCO between both groups observed. Dm diminished also significantly in both groups during treatment, but differences between both groups were not seen. VC and Vc decreased in patients receiving BEP but remained constant during treatment with EP. It can be concluded that the Dm, KCO, and the widely used TLCO are not suitable parameters to monitor specifically pulmonary toxicity induced by bleomycin as part of a multidrug regimen. However, VC and Vc appear to be proper lung function assessments which reflect specifically alterations induced by bleomycin.


### Decrease in pulmonary function during bleomycin-containing combination chemotherapy for testicular cancer: not only a bleomycin effect

S Sleijfer[1], TW van der Mark[2], H Schraffordt Koops[3] and NH Mulder[1]

*Divisions of [1]Medical Oncology and [2]Lung Function of the Department of Internal Medicine and [3]Department of Surgical Oncology, University Hospital Groningen, Groningen, Oostersingel 59, 9713 EZ Groningen, The Netherlands.*

**Summary** This study was performed to determine the changes in pulmonary function in patients randomised to receive treatment with four cycles of bleomycin, etoposide and cisplatin (BEP) (27 patients) or with four cycles of etoposide and cisplatin (EP) (27 patients) for disseminated non-seminomatous testicular cancer. This enabled us to establish whether effects other than those due to bleomycin determined the detrimental effects of BEP on lung function assessments. Slow inspiratory vital capacity (VC), the transfer factor of the lungs for carbon monoxide ($T_{LCO}$), the diffusing capacity of the alveolo-capillary membrane ($D_m$), the pulmonary capillary blood volume ($V_c$) and the transfer factor of the lungs for carbon monoxide per unit alveolar volume ($K_{CO}$) were determined before and at 3 week intervals during chemotherapy. Both groups, similar in terms of factors that may influence pulmonary function, showed during therapy a significant decrease in $T_{LCO}$ compared with the pretreatment value. Only at the end of the therapy was a significant difference in $T_{LCO}$ between both groups observed. $D_m$ diminished also significantly in both groups during treatment, but differences between both groups were not seen. VC and $V_c$ decreased in patients receiving BEP but remained constant during treatment with EP. It can be concluded that the $D_m$, $K_{CO}$, and the widely used $T_{LCO}$ are not suitable parameters to monitor specifically pulmonary toxicity induced by bleomycin as part of a multidrug regimen. However, VC and $V_c$ appear to be proper lung function assessments which reflect specifically alterations induced by bleomycin.

**Keywords:** bleomycin; cisplatin; pulmonary function; testicular cancer

The standard chemotherapy for patients with disseminated testicular germ cell tumours consists of a combination of bleomycin, etoposide and cisplatin (BEP) (Williams *et al.*, 1987), leading to an overall 5 year survival of these patients of 87% (Dearnaley *et al.*, 1991). A major disadvantage of this treatment is the occurrence of bleomycin-induced pneumonitis (BIP). The reported incidence of this toxicity varies from 0 to 46% of the patients receiving a bleomycin-containing therapy (De Lena *et al.*, 1972; Van Barneveld *et al.*, 1984; Williams *et al.*, 1987; Jules-Elysee *et al.*, 1990; Dearnaley *et al.*, 1991; Osanto *et al.*, 1992). Fatal BIP occurs in approximately 3% of patients treated with bleomycin (Levi *et al.*, 1993). However, bleomycin nowadays is only used as part of a multidrug chemotherapeutic regimen, and so the impact of bleomycin alone on lung function is not known.

The transfer factor of the lungs for carbon monoxide ($T_{LCO}$) is assumed to reflect alterations in the lungs caused by bleomycin, and therefore $T_{LCO}$ has become a tool to detect BIP (Comis, 1992). The value of $T_{LCO}$ is determined by the diffusing capacity of the alveolo-capillary membrane for carbon monoxide ($D_m$) and the pulmonary capillary blood volume ($V_C$). Previously, we have shown that determination of $T_{LCO}$, and especially of the two components of $T_{LCO}$, $D_m$ and $V_C$, and determination of the slow inspiratory vital capacity (VC) are the best indicators of pulmonary toxicity during a bleomycin-containing treatment for disseminated testicular cancer (Luursema *et al.*, 1983; Van Barneveld *et al.*, 1985). Moreover, we showed that when BIP occurs it is completely reversible with time (Van Barneveld *et al.*, 1987).

In recent years, different prognostic systems have been developed in order to predict the outcome of therapy in patients with disseminated testicular germ cell tumours. Based on these systems, patients can be divided into groups with a good or a poor prognosis. For the treatment of patients with a good prognosis less toxic chemotherapy regimens are studied. Therefore, the EORTC Genito-Urinary Tract Group started a randomised study comparing BEP with etoposide and cisplatin (EP) in good-prognosis patients (Stoter *et al.*, 1987). We have evaluated as a side study the influences of BEP as well as of EP on the pulmonary function parameters mentioned above and the transfer factor of the lungs for carbon monoxide per unit alveolar volume ($K_{CO}$). This gave us the opportunity to study the detrimental effects of bleomycin and the other drugs of the combination separately and to establish whether the lung function assessments examined are suitable parameters which reflect specifically changes induced by bleomycin.

### Patients and methods

Fifty-four patients with low-volume metastases of a disseminated non-seminomatous tumour of the testis were randomised. Low-volume metastases were defined as infradiaphragmatic nodal disease smaller than 5 cm diameter, mediastinal nodal disease smaller than 5 cm diameter, supraclavicular nodal disease smaller than 5 cm diameter or lung metastases four or less in number and smaller than 2 cm in diameter.

Twenty-seven patients, mean age 31 years (range 21-44), were treated with four cycles of BEP, consisting of cisplatin 20 mg $m^{-2}$ i.v. on days 1-5, every 3 weeks, etoposide 120 mg $m^{-2}$ i.v. on days 1, 3 and 5 every 3 weeks and bleomycin 30 mg dissolved in 100 ml of 0.9% sodium chloride intravenously infused over 15 min on day 2 and thereafter weekly for 12 weeks.

Twenty-seven patients, mean age 31 years (range 17-51), were treated with four cycles of EP, consisting of cisplatin and etoposide only.

Patient records were checked for factors known to influence the lung function parameters examined, such as previous pulmonary disease (e.g. asthma) and smoking habits. Before the start of the chemotherapy and at 3 week intervals during treatment, lung function tests were carried out.

Slow inspiratory vital capacity (VC) was measured with a standard water-sealed spirometer. $T_{LCO}$ was measured with the single-breath technique of Krogh *et al.* (1914) modified

Correspondence: NH Mulder

Received 10 June 1994; revised 29 July 1994; accepted 2 August 1994

by Ogilvie et al. (1957) and Cotes (1979). $D_m$ and $V_C$ were determined by measuring $T_{LCO}$ at two different oxygen concentrations (18.4% and 88.0%). The calculations were performed according to the equation given by Roughton et al. (1957). All measurements were carried out in duplicate. The values of $T_{LCO}$ have been corrected for the haemoglobin concentration according to Cotes (1979) in order to obtain a value of $T_{LCO}$ under standard conditions. The alveolar volume ($V_A$) was calculated from the inspiratory and expiratory helium concentrations measured during the determination of $T_{LCO}$. The transfer factor of the lungs for carbon monoxide per unit alveolar volume ($K_{CO}$) was obtained by dividing the $T_{LCO}$, corrected for haemoglobin concentration, by the alveolar volume. Lung functions were expressed as a percentage of the predicted value according to the regression equations given by Cotes (1979).

BIP was defined as a clinical syndrome featuring dry cough, exertional dyspnoea, dyspnoea at rest, tachypnoea, fever and cyanosis. On chest radiography, BIP was revealed by a fine reticular bibasilar infiltrate, an alveolar interstitial bibasilar infiltrate, progressive lower lobe involvement or lobar consolidation (Comis, 1992). Bleomycin was discontinued in those patients developing BIP. Changes in lung function parameters were not used to reduce bleomycin dosages, nor was dose reduction allowed for any other reason. Statistical analysis to compare changes in lung functions to pretreatment values was performed by analysis of variance (ANOVA) in a repeated measurement design. For analysing differences in lung and renal functions between both therapies, two-tailed unpaired Student t-tests were used. P-values below 0.05 were considered to be significant.

### Results

The characteristics of the patients are summarised in Table I. Factors that may influence the course of the pulmonary functions such as age, smoking habits and changes in renal function (creatinine clearance) during treatment did not differ between both groups of patients. The incidence of prior lung disease was negligible, and no patients received oxygen therapy or previous anti-cancer therapy. In the group treated with BEP, there were more patients with lung metastases. However, pretreatment values of lung function did not differ between patients with or without lung metastases (results not shown).

Of the 27 patients treated with BEP, three developed BIP, all in the fourth cycle of therapy (weeks 9–12). These patients were not given the twelfth administration of bleomycin and so received 330 mg of bleomycin in total, in contrast to the other patients in the BEP group, who received 360 mg in total. The lung function parameters of the patients who developed BIP did not differ significantly from the other patients treated with BEP (results not shown). Among the patients who received EP, none developed pulmonary symptoms.

In both the BEP group and the EP group, $T_{LCO}$ decreased compared with pretreatment values (Figure 1). This decline was significant in both groups in week 6 ($P<0.01$). This decline continued, and the difference from the pretreatment value increased until week 12 ($P<0.001$). A significant difference between both groups was only observed in week 12 ($P<0.01$) when the decrease in the BEP group was most prominent.

The pulmonary capillary blood volume ($V_C$), one of the components of the $T_{LCO}$, did not change in the EP group during treatment (Figure 2). However, in the BEP group the $V_C$ decreased significantly from week 9 onwards ($P<0.05$). Compared with the EP group, the $V_C$ in the BEP group was significantly lower in weeks 9 and 12 ($P<0.05$).

Figure 3 shows that the other component of $T_{LCO}$, the diffusing capacity of the alveolo-capillary membrane ($D_m$), diminished in both groups. Compared with the pretreatment value, this decrease was significant in the BEP group from weeks 9 to 12 ($P<0.01$) and in the EP group from week 6 until the end ($P<0.001$). The reduction in $D_m$ seemed to lessen in the EP group during the last 3 weeks of treatment. $D_m$ values did not differ significantly between the groups.

The course of $K_{CO}$, $T_{LCO}$ corrected for differences in alveolar volume ($V_A$), is depicted in Figure 4. $K_{CO}$ decreased in the BEP group as well as in the EP group, and this decline was significant in both groups from weeks 3 to 12 (week 3, $P<0.05$; weeks 6–12, $P<0.001$). No difference was observed between therapies.

The VC had a tendency to increase during treatment with EP, but this increase did not reach significance (Figure 5). In the BEP group, however, VC increased significantly from the start to week 3 of treatment ($P<0.05$). Thereafter, the VC declined, and this was significant in week 12 compared with the pretreatment value ($P<0.01$). Only in week 12 was a significant difference in VC between both groups observed ($P<0.01$).

Table I Patients' characteristics

| | BEP | EP |
|---|---|---|
| No. of patients | 27 | 27 |
| Mean age in years (range) | 31 (21–44) | 31 (17–51) |
| Lung metastases (no. of patients) | 8 | 3 |
| Smoking history (no. of patients) | 9 | 11 |
| Mean creatinine clearance in ml min$^{-1}$ (s.d.) before cycle | | |
| 1 | 145 (26) | 136 (24) |
| 2 | 143 (26) | 132 (27) |
| 3 | 139 (26) | 131 (29) |
| 4 | 126 (26) | 128 (20) |

Figure 1 Changes in $T_{LCO}$ in patients treated with BEP (■) or EP (▲) (per cent mean difference from pretreatment value). Error bars represent the standard deviation.

Figure 2 Changes in $V_C$ in patients treated with BEP (■) or EP (▲) (per cent mean difference from pretreatment value). Error bars represent the standard deviation.

**Figure 3** Changes in $D_m$ in patients treated with BEP (■) or EP (▲) (per cent mean difference from pretreatment value). Error bars represent the standard deviation.

**Figure 4** Changes in $K_{CO}$ in patients treated with BEP (■) or EP (▲) (per cent mean difference from pretreatment value). Error bars represent the standard deviation.

**Figure 5** Changes in VC in patients treated with BEP (■) or EP (▲) (per cent mean difference from pretreatment value). Error bars represent the standard deviation.

### Discussion

Although bleomycin has been successful as part of a combination with etoposide or vinblastine and cisplatin against germ cell cancer, bleomycin is suspected of inducing pulmonary toxicity. To avoid this toxicity, the EORTC Genito-Urinary Tract Group started a randomised study to compare the anti-tumour efficacy and toxicity of treatment with BEP compared with the probably less toxic treatment with EP (Stoter *et al.*, 1987). We performed a side study to monitor the changes in vital capacity (VC), transfer factor of the lungs for carbon monoxide ($T_{LCO}$), the diffusing capacity of the alveolo-capillary membrane ($D_m$), the pulmonary capillary blood volume ($V_C$) and the transfer factor for carbon monoxide per unit alveolar volume ($K_{CO}$) during treatment with BEP or EP in all patients entering this study in our centre.

Based on histological changes in animals observed after administration of bleomycin (Adamson *et al.*, 1974), it was assumed that the reduction in $T_{LCO}$ observed during treatment of patients with bleomycin-containing combination chemotherapy was predominantly caused by the bleomycin. Moreover, a decrease in $T_{LCO}$ below the 60% pretreatment value has been used as an argument to reduce or to discontinue the doses of bleomycin administered during treatment (Comis *et al.*, 1979; Ginsberg *et al.*, 1982). However, bleomycin is used not as a single agent but almost always in combination with other chemotherapeutic drugs. Thus, changes in $T_{LCO}$ during treatment may also be caused by the other agents used such as etoposide/vinblastine and cisplatin.

The change in $T_{LCO}$ found in this study during treatment with BEP is consistent with previous studies of bleomycin-containing therapy performed by us and others (Comis *et al.*, 1979; Luursema *et al.*, 1983; Sørensen *et al.*, 1985; Van Barneveld *et al.*, 1985; White *et al.*, 1987; Hansen *et al.*, 1989; Wolkowicz *et al.*, 1992). However, our study reveals that this decline is not caused only by bleomycin because the $T_{LCO}$ also decreased in patients treated with EP only. Moreover, the continuing decrease in $T_{LCO}$ during treatment with BEP and EP only differs at the very end of therapy when the decline in $T_{LCO}$ is most pronounced in the BEP group and reached significant difference. This difference is probably due to a reduction in the alveolar volume ($V_A$) in the BEP group compared with the EP group, because no obvious differences are observed between the groups when the $T_{LCO}$ is corrected for changes in $V_A$ by applying the $K_{CO}$. The decline in $T_{LCO}$ in the EP group is accompanied by a decrease in $D_m$, while $V_C$ remains constant. Thus, it can be concluded that the reduction in $T_{LCO}$ is caused by alterations in the alveolo-capillary membrane induced by etoposide and/or cisplatin and that these two agents do not have any effect on the $V_C$. How etoposide and/or cisplatin induce these changes which lead to decreases in both $D_m$ and $T_{LCO}$ is not known. Cisplatin has not previously been reported to induce such changes in lung functions, and etoposide-related pulmonary toxicity is only very sporadically described (Zimmerman *et al.*, 1984). In contrast, capillary alterations caused by these agents are well known and recently reviewed by Doll *et al.* (1992).

Because the decrease in $D_m$ is also observed in the BEP group, it can be assumed that bleomycin has only a minor impact on $D_m$ and that this decline is also due to effects of etoposide and /or cisplatin. As Van Barneveld *et al.* (1985) observed a reduction in $D_m$ in patients treated with cisplatin, vinblastine and bleomycin, it is conceivable that cisplatin is the major cause of this decline. The reduction in $V_C$, which only occurs in patients receiving bleomycin, indicates that bleomycin does have an effect on the lung vasculature, but different from the effect of etoposide and cisplatin. The capillary changes observed using nailfold capillary microscopic examination (Bellmunt *et al.*, 1987), the reported cases of Raynaud's phenomenon (Vogelzang *et al.*, 1981; Adoue *et al.*, 1984), myocardial infarction (Samuels *et al.*, 1987) and pulmonary veno-occlusive disease (Joselson *et al.*, 1983) induced by bleomycin alone or in combination therapy also suggest direct effects of bleomycin on the vasculature. The effects of bleomycin occur predominantly in the lung and skin vasculature, because these two organs are, in contrast to other organs such as the liver, deficient in bleomycin hydrolase which inactivates bleomycin (Ohnuma *et al.*, 1974).

Bleomycin-related alterations of the vasculature are due to induction of free radicals by bleomycin, which causes endothelial damage, followed by an immunological process with infiltration of lymphocytes and alveolar macrophages (Adamson *et al.*, 1974). These cells produce cytokines, which further augment cellular accumulation and cause, in combination with bleomycin, proliferation of fibroblasts (Moseley *et al*., 1986), production of collagen (Otsuka *et al*., 1978) and finally fibrosis (Khalil *et al*., 1989), which may cause occlusion of capillaries, which probably leads to the observed reduction in $V_C$ in the patients treated with BEP.

Van Barneveld *et al*. (1985) have shown in patients with testicular cancer a decline in VC during chemotherapy consisting of bleomycin, vinblastine and cisplatin. This study confirms these results, using etoposide instead of vinblastine, and shows that this decline in VC is specifically caused by bleomycin, because this effect only occurs in patients treated with BEP.

The observation that the lung function assessments of the three patients who developed BIP did not differ significantly from the other patients receiving BEP does not confirm the results of Van Barneveld *et al*. (1985), although three patients is too small a number to draw conclusions about the usage of the function tests examined for monitoring BIP.

In conclusion, this study shows that the $T_{LCO}$, widely used to monitor the potential lethal pulmonary toxicity induced by bleomycin as part of a multidrug anti-cancer treatment, is not a proper parameter for this purpose as it actually might measure effects of etoposide and cisplatin. Also, changes in the diffusing capacity of the alveolo-capillary membrane ($D_m$) and $K_{CO}$ are not specific for detecting bleomycin-induced toxicity. In contrast, bleomycin induces pulmonary alterations that are specifically reflected in the pulmonary capillary blood volume ($V_C$) and vital capacity (VC). These two parameters may be the most suitable for determining bleomycin effects in the lung.

### References

ADAMSON IYR AND BOWDEN DH. (1974). The pathogenesis of bleomycin-induced pulmonary fibrosis in mice. *Am. J. Pathol.*, **77**, 185–191.

ADOUE D AND ARLET P. (1984). Bleomycin and Raynaud's phenomenon (letter). *Ann. Int. Med.*, **100**, 770.

BELLMUNT J, NAVARRO M, MORALES S, JOLIS L, CARULLA J, KNOBELL H, VILARDEL M AND SOLE LA. (1987). Capillary microscopy is a potentially useful method for detecting bleomycin vascular toxicity. *Cancer*, **65**, 253–256.

COMIS RL. (1992). Bleomycin pulmonary toxicity: current status and future decisions. *Semin. Oncol.*, **19** (Suppl. 5), 64–70.

COMIS RL, KUPPINGER MS, GINSBERG SJ, CROOKE ST, GILBERT R, AUCHINCLOSS JH AND PRESTAYKO AW. (1979). Role of single-breath carbon monoxide diffusing capacity in monitoring the pulmonary effects of bleomycin in germ cell tumor patients. *Cancer Res.*, **39**, 5076–5080.

COTES JE. (1979). *Lung Function*. Blackwell Scientific Publications: Boston.

DEARNALEY DP, HORWICH A, A'HERN R, NICHOLLS J, JAY G, HENDRY WF AND PECKHAM MJ. (1991). Combination chemotherapy with bleomycin, etoposide and cisplatin (BEP) for metastatic testicular teratoma: long-term follow-up. *Eur. J. Cancer*, **27**, 684–691.

DE LENA M, GUZZON A, MONFARDINI S AND BONADONNA G. (1972). Clinical, radiologic and histopathological studies on pulmonary toxicity induced by treatment with bleomycin (N.S.C.-125066). *Cancer Chemother. Rep.*, **56**, 343–355.

DOLL DC AND YARBO JW. (1992). Vascular toxicity with neoplastic agents. *Semin. Oncol.*, **19**, 580–596.

GINSBERG SJ AND COMIS RL. (1982). The pulmonary toxicity of antineoplastic agents. *Semin. Oncol.*, **9**, 34–51.

HANSEN SW, GROTH S, SØRENSEN PG, ROSSING N AND RØRTH M. (1989). Enhanced pulmonary toxicity in smokers with germ-cell cancer treated with cisplatinum, vinblastine and bleomycin: a long term follow-up. *Eur. J. Cancer Clin. Oncol.*, **25**, 733–736.

JOSELSON R AND WARNOCK M. (1983). Pulmonary veno-occlusive disease after chemotherapy. *Hum. Pathol.*, **13**, 88–91.

JULES-ELYSEE K AND WHITE DA. (1990). Bleomycin-induced pulmonary toxicity. *Clin. Chest Med.*, **11**, 1–20.

KHALIL N, BEREZNAY O, SPORN M AND GREENBERG AH. (1989). Macrophage production of transforming growth factor $\beta$ and fibroblast collagen synthesis in chronic pulmonary inflammation. *J. Exp. Med.*, **170**, 727–737.

KROGH M. (1914). The diffusion of gases through the lungs of man. *J. Appl. Physiol.*, **49**, 271–300.

LEVI JA, RAGHAVAN D, HARVEY V, THOMPSON D, SANDEMAN T, GILL G, STUART-HARRIS R, SNYDER R, BYRNE M, KERESTES Z AND MARGRIE S. (1993). The importance of bleomycin in combination chemotherapy for good-prognosis germ cell carcinoma. *J. Clin. Oncol.*, **11**, 1300–1305.

LUURSEMA PB, STAR-KROESEN MA, VAN DER MARK THW, SLEIJFER DTH, SCHRAFFORDT KOOPS H, SLUITER HJ AND PESET R. (1983). Bleomycin induced changes in the carbon monoxide transfer factor of the lung and its components. *Am. Rev. Respir. Dis.*, **128**, 880–883.

MOSELEY PL, HEMKEN C AND HUNNINGHAKE GW. (1986). Augmentation of fibroblast proliferation by bleomycin. *J. Clin. Invest.*, **78**, 1150–1154.

OGILVIE CM, FORSTER RE, BLANEMORE WS AND MARSON JWA. (1957). Standardized breath holding technique for the clinical measurement of the lung for carbon monoxide. *J. Clin. Invest.*, **36**, 1–17.

OHNUMA T, HOLLAND JF, MASUDA H, WALIGUNDA JA AND GOLDBERG GA. (1974). Microbiological assay of bleomycin: inactivation, tissue distribution, and clearance. *Cancer*, **33**, 1230–1238.

OSANTO S, BUKMAN A, VAN HOEK F, STERK PJ, DE LAAT JAPM AND HERMANS J. (1992). Long term effects of chemotherapy in patients with testicular cancer. *J. Clin. Oncol.*, **10**, 574–579.

OTSUKA K, MUROTA S AND MORI Y. (1978). Stimulatory effect of bleomycin on the hyaluronic acid synthetase in cultured fibroblasts. *Biochem. Pharmacol.*, **27**, 1551–1554.

ROUGHTON FJW AND FORSTER RE. (1957). Relative importance of diffusion and chemical reaction rates in determining rate of exchange of gases in the human lung, with special reference to true diffusing capacity of pulmonary membrane and volume of blood in the lung capillaries. *J. Appl. Physiol.*, **11**, 290–302.

SAMUELS BL, VOGELZANG NJ AND KENNEDY BJ. (1987). Severe vascular toxicity associated with vinblastine, bleomycin, and cisplatin chemotherapy. *Cancer Chemother. Pharmacol.*, **19**, 253–256.

SØRENSEN PG, ROSSING N AND RØRTH M. (1985). Carbon monoxide diffusion capacity: a reliable indicator of bleomycin induced pulmonary toxicity. *Eur. J. Respir. Dis.*, **66**, 333–340.

STOTER G, KAYE S, JONES W, TEN BOKKEL-HUININK W, SLEIJFER D, VAN OOSTEROM A, HARRIS A, BOVEN E, DE PAUW M AND SYLVESTER R. (1987). Cisplatin and VP-16 +/− bleomycin (BEP vs EP) in good risk patients with disseminated non-seminomatous testicular cancer: a randomized EORTC GU Group study. *Proc. Am. Soc. Clin. Oncol.*, **6**, 110.

VAN BARNEVELD PWC, VAN DER MARK THW, SLEIJFER DTH, MULDER NH, SCHRAFFORDT KOOPS H, SLUITER HJ AND PESET R. (1984). Predictive factors for bleomycin-induced pneumonitis. *Am. Rev. Respir. Dis.*, **130**, 1078–1081.

VAN BARNEVELD PWC, VEENSTRA G, SLEIJFER DTH, VAN DER MARK THW, MULDER NH, SCHRAFFORDT KOOPS H, SLUITER HJ AND PESET R. (1985). Changes in pulmonary functions during and after bleomycin treatment in patients with testicular carcinoma. *Cancer Chemother. Pharmacol.*, **14**, 168–171.

VAN BARNEVELD PWC, SLEIJFER DTH, VAN DER MARK THW, MULDER NH, SCHRAFFORDT KOOPS H, SLUITER HJ AND PESET R. (1987). The natural course of bleomycin induced pneumonitis (BIP) – a follow up study in eight patients. *Am. Rev. Respir. Dis.*, **135**, 48–51.

VOGELZANG NJ, BOSL GJ, JOHNSON K AND KENNEDY BJ. (1981). Raynaud's phenomenon: a common toxicity after combination chemotherapy for testicular cancer. *Ann. Int. Med.*, **95**, 288–292.

WHITE DA, STOVER DE, SMITH G AND BECK G. (1987). Serial pulmonary function studies during bleomycin therapy. *Am. Rev. Respir. Dis.*, **135** (Suppl.), A39.

WILLIAMS SD, BIRCH R, EINHORN LH, IRWIN L, GRECO FA AND LOEHRER PJ. (1987). Treatment of disseminated germ-cell tumors with cisplatin, bleomycin, and either vinblastine or etoposide. *N. Engl. J. Med.*, **316**, 1435–1440.

WOLKOWICZ J, STURGEON J, RAVJI M AND CHAN CK. (1992). Bleomycin induced pulmonary function abnormalities. *Chest*, **101**, 97–101.

ZIMMERMAN MS, RUCKDESCHEL JC AND HUSSAIN M. (1984). Chemotherapy-induced interstitial pneumonitis during treatment of small cell anaplastic lung cancer. *J. Clin. Oncol.*, **2**, 396–405.